# E-cadherin breast tumor expression, risk factors and survival: Pooled analysis of 5,933 cases from 12 studies in the Breast Cancer Association Consortium

**DOI:** 10.1038/s41598-018-23733-4

**Published:** 2018-04-26

**Authors:** Hisani N. Horne, Hannah Oh, Mark E. Sherman, Maya Palakal, Stephen M. Hewitt, Marjanka K. Schmidt, Roger L. Milne, David Hardisson, Javier Benitez, Carl Blomqvist, Manjeet K. Bolla, Hermann Brenner, Jenny Chang-Claude, Renata Cora, Fergus J. Couch, Katarina Cuk, Peter Devilee, Douglas F. Easton, Diana M. Eccles, Ursula Eilber, Jaana M. Hartikainen, Päivi Heikkilä, Bernd Holleczek, Maartje J. Hooning, Michael Jones, Renske Keeman, Arto Mannermaa, John W. M. Martens, Taru A. Muranen, Heli Nevanlinna, Janet E. Olson, Nick Orr, Jose I. A. Perez, Paul D. P. Pharoah, Kathryn J. Ruddy, Kai-Uwe Saum, Minouk J. Schoemaker, Caroline Seynaeve, Reijo Sironen, Vincent T. H. B. M. Smit, Anthony J. Swerdlow, Maria Tengström, Abigail S. Thomas, A. Mieke Timmermans, Rob A. E. M. Tollenaar, Melissa A. Troester, Christi J. van Asperen, Carolien H. M. van Deurzen, Flora F. Van Leeuwen, Laura J. Van’t Veer, Montserrat García-Closas, Jonine D. Figueroa

**Affiliations:** 1 Division of Cancer Epidemiology and Genetics, National Cancer Institute, Rockville, MD USA; 2 Division of Molecular Genetics & Pathology, US Food and Drug Administration, Silver Spring, MD USA; 3 Department of Health Policy and Management, College of Health Science, Korea University, Seoul, Korea; 4 Health Sciences Research, Mayo Clinic, Jacksonville, FL USA; 5 Center for Cancer Research, National Cancer Institute, Bethesda, MD USA; 6 Division of Molecular Pathology, The Netherlands Cancer Institute - Antoni van Leeuwenhoek Hospital, Amsterdam, The Netherlands; 7 Division of Psychosocial Research and Epidemiology, The Netherlands Cancer Institute - Antoni van Leeuwenhoek hospital, Amsterdam, The Netherlands; 8 Cancer Epidemiology & Intelligence Division, Cancer Council Victoria, Melbourne, Victoria, Australia; 9 Centre for Epidemiology and Biostatistics, Melbourne School of Population and Global health, The University of Melbourne, Melbourne, Victoria, Australia; 10 Department of Pathology, Molecular Pathology and Therapeutic Targets Group, Hospital Universitario La Paz IdiPAZ, and Facultad de Medicina, Universidad Autonoma de Madrid, Madrid, Spain; 11 Human Cancer Genetics Program, Spanish National Cancer Research Centre, Madrid, Spain; 12 Centro de Investigación en Red de Enfermedades Raras (CIBERER), Valencia, Spain; 13 Department of Oncology, Helsinki University Hospital, University of Helsinki, Helsinki, Finland; 14 Centre for Cancer Genetic Epidemiology, Department of Public Health and Primary Care, University of Cambridge, Cambridge, UK; 15 Division of Clinical Epidemiology and Aging Research, German Cancer Research Center (DKFZ), Heidelberg, Germany; 16 Division of Preventive Oncology, German Cancer Research Center (DKFZ) and National Center for Tumor Diseases (NCT), Heidelberg, Germany; 17 German Cancer Consortium (DKTK), German Cancer Research Center (DKFZ), Heidelberg, Germany; 18 Division of Cancer Epidemiology, German Cancer Research Center (DKFZ), Heidelberg, Germany; 19 Research Group Genetic Cancer Epidemiology, University Cancer Center Hamburg (UCCH), University Medical Center Hamburg-Eppendorf, Hamburg, Germany; 20 Independent contractor, CT(ASCP), MB (ASCP), National Cancer Institute, Bethesda, MD USA; 21 Department of Laboratory Medicine and Pathology, Mayo Clinic, Rochester, MN USA; 22 Department of Pathology, Leiden University Medical Center, Leiden, The Netherlands; 23 Department of Human Genetics, Leiden University Medical Center, Leiden, The Netherlands; 24 Centre for Cancer Genetic Epidemiology, Department of Oncology, University of Cambridge, Cambridge, UK; 25 Cancer Sciences Academic Unit, Faculty of Medicine, University of Southampton, Southampton, UK; 26 Translational Cancer Research Area, University of Eastern Finland, Kuopio, Finland; 27 Institute of Clinical Medicine, Pathology and Forensic Medicine, University of Eastern Finland, Kuopio, Finland; 28 Imaging Center, Department of Clinical Pathology, Kuopio University Hospital, Kuopio, Finland; 29 Department of Pathology, Helsinki University Hospital, University of Helsinki, Helsinki, Finland; 30 Saarland Cancer Registry, Saarbrücken, Germany; 31 Department of Medical Oncology, Family Cancer Clinic, Erasmus MC Cancer Institute, Rotterdam, The Netherlands; 32 Division of Genetics and Epidemiology, The Institute of Cancer Research, London, UK; 33 Department of Obstetrics and Gynecology, Helsinki University Hospital, University of Helsinki, Helsinki, Finland; 34 Department of Health Sciences Research, Mayo Clinic, Rochester, MN, USA; 35 The Breast Cancer Now Toby Robins Research Centre, The Institute of Cancer Research, London, UK; 36 Servicio de Cirugía General y Especialidades, Hospital Monte Naranco, Oviedo, Spain; 37 Division of Breast Cancer Research, The Institute of Cancer Research, London, UK; 38 Cancer Center, Kuopio University Hospital, Kuopio, Finland; 39 Institute of Clinical Medicine, Oncology, University of Eastern Finland, Kuopio, Finland; 40 Department of Surgery, Leiden University Medical Center, Leiden, The Netherlands; 41 Department of Pathology and Laboratory Medicin, Gillings School of Global Public Health, Lineberger Comprehensive Cancer Center, University of North Carolina, Chapel Hill, NC USA; 42 Department of Clinical Genetics, Leiden University Medical Center, Leiden, The Netherlands; 43 Department of Pathology, Erasmus University Medical Center, Rotterdam, The Netherlands; 44 Usher Institute of Population Health Sciences and Informatics, The University of Edinburgh Medical School, Edinburgh, UK; 45 Department of Oncology, Mayo Clinic, Rochester, MN USA

## Abstract

E-cadherin (CDH1) is a putative tumor suppressor gene implicated in breast carcinogenesis. Yet, whether risk factors or survival differ by E-cadherin tumor expression is unclear. We evaluated E-cadherin tumor immunohistochemistry expression using tissue microarrays of 5,933 female invasive breast cancers from 12 studies from the Breast Cancer Consortium. H-scores were calculated and case-case odds ratios (OR) and 95% confidence intervals (CIs) were estimated using logistic regression. Survival analyses were performed using Cox regression models. All analyses were stratified by estrogen receptor (ER) status and histologic subtype. E-cadherin low cases (N = 1191, 20%) were more frequently of lobular histology, low grade, >2 cm, and HER2-negative. Loss of E-cadherin expression (score < 100) was associated with menopausal hormone use among ER-positive tumors (ever compared to never users, OR = 1.24, 95% CI = 0.97–1.59), which was stronger when we evaluated complete loss of E-cadherin (i.e. H-score = 0), OR = 1.57, 95% CI = 1.06–2.33. Breast cancer specific mortality was unrelated to E-cadherin expression in multivariable models. E-cadherin low expression is associated with lobular histology, tumor characteristics and menopausal hormone use, with no evidence of an association with breast cancer specific survival. These data support loss of E-cadherin expression as an important marker of tumor subtypes.

## Introduction

The E-cadherin protein (encoded by the *CDH1* gene) is normally expressed in breast epithelial tissue and functions as a critical part of epithelial cell adhesion and epithelial-to-mesenchymal transition (EMT)^1–3^. Due to the frequent loss or inactivation of E-cadherin that is evident in epithelial cell cancers, E-cadherin is thought to have tumor-suppressor properties where loss is associated with carcinogenesis and invasion^4,5^.

Loss of E-cadherin expression is commonly used to confirm lobular histology that comprise 10–15% of all breast cancers^6–8^, which have also been noted to more frequently express hormone receptors [estrogen receptor (ER) and progesterone receptor (PR)] than E-cadherin high tumors^9^. Recent molecular profiling analysis of lobular compared with ductal cancers show E-cadherin mutation and loss to be a defining feature of lobular breast cancers, and suggest it to be a distinct molecular subtype of breast cancers^10^. Although many studies have identified heterogeneity in risk factor associations based on breast tumor subtypes defined by hormone receptor status (e.g. ER-positive vs. ER-negative) or histology (lobular vs. ductal)^11–22^, limited data have examined whether E-cadherin may define important subgroups of tumors with distinct etiologies^23^.

Data also suggest that loss of E-cadherin expression may be associated with malignant progression, metastasis, and reduced survival in breast cancer patients^24–28^; however, most of these studies were based on small numbers and not all analyses were stratified by ER-status, a known important prognostic and predictive marker.

Evidence on whether E-cadherin may be an important marker of etiologic heterogeneity and survival differences across the spectrum of breast tumor subtypes including ER status and histology is limited. In 1984, Prentice *et al*.^29^ introduced the concept of using case-case studies for identifying disease risk factors. In a case-case study design, information is obtained only from cases of a particular disease–in our study, breast cancer–and is used as a tool to assess etiologic heterogeneity. In this study, we were interested in determining whether known risk/protective factors for breast cancer differed by E-cadherin expression, in order to provide new insights into possible mechanisms for E-cadherin loss in breast carcinogenesis similar to analyses previously done for ER, PR and HER2 markers^23,30,31^. Using immunohistochemical (IHC) data for E-cadherin performed centrally using tumor tissue microarrays (TMAs), we performed a large pooled analysis of 12 studies participating in the Breast Cancer Association Consortium (BCAC), and examined whether established breast cancer risk factor associations and survival differed by low vs. high E-cadherin tumor tissue expression, stratified by ER status and histology.

## Material and Methods

### Study Population

Descriptions of the 12 breast cancer studies participating in the Breast Cancer Association Consortium (BCAC) included in this analysis are detailed in Supplemental Table 1. Case-case analyses were restricted to 5,933 European women from 12 breast cancer studies with invasive breast cancer who provided data on age at diagnosis and had evaluable E-cadherin tumor tissue staining results (See section on E-cadherin tumour tissue measurements). Study participants were recruited under protocols approved by the Institutional Review Board at each institution, and all subjects provided informed consent or did not opt-out, depending on national regulations. All methods were performed in accordance with the relevant guidelines and regulations and a list of ethical approval committees are listed at the end of this manuscript.

### Risk factor information

The 12 participating studies provided information on one or more of the following risk factors for breast cancer: family history of breast cancer in first-degree relatives, reproductive factors including age at menarche, parity, age at first full-term birth, oral contraceptive (OC) use among women ≤50 years of age, menopausal hormonal use, type of ever menopausal hormone used, and anthropometric measures including body mass index (BMI), and height. As a proxy for menopausal status, we used age ≤50 and >50 as a proxy for pre- and postmenopausal status respectively, since not all studies captured menopausal status.

### E-cadherin tumor tissue measurements

Routinely prepared formalin-fixed paraffin-embedded (FFPE) blocks of invasive breast tumors were used to construct TMA blocks at each study center. One-hundred and forty-two TMA slides with tumor samples from 6,010 individual patients were prepared for E-cadherin staining (ranging from 1–2 cores per patient). TMA’s from all participating studies were stained centrally in the Experimental Pathology Laboratory at the National Cancer Institute (NCI) to allow for consistency across sites and avoid any potential batch effect that may arise due to systematic variation in staining procedures. We recognized the study is unable to control for pre-analytic variables in tissue fixation and processing. However, to address this, the Experimental Pathology Lab at NCI carefully re-titrated the IHC assay to provide a stable assay across all samples.

IHC staining was performed on a Benchmark ULTRA autostainer (Ventana Medical Systems, Tuscon, AZ). TMA sections were deparaffinized with zylene and graded alcohols; antigen retrieval was mediated with citrate buffer pH 9 (Dako) for 20 minutes in a pressure cooker. Primary mouse monoclonal antibody, anti-E-cadherin (clone NCH-38, 1:500; Dako, Carpinteria, CA) was applied at room temperature for 2 hours. The antigen-antibody complex was detected using Envision + (Dako) and DAB was applied for 20 minutes. Slides were counterstained with hematoxylin, dehydrated and coverslipped. Slides were imaged with a Hamamatsu Nanozoomer (Bridgewater, NJ), at 20× magnification and cataloged using the SlidePath Digital Image Hub (Leica Biosystems, Wetzlar, Germany).

As our primary interest was in investigating clinically relevant expression of E-cadherin expression, we used the H-scoring system as has been proposed and evaluated in previous publications^23–25^. Two cytotechnologists assessed digital images of TMA spots using the SlidePath Digital Image Hub, blinded to any clinical data. Manual readings of each TMA spot recorded the quality of the image (unsatisfactory, limited or satisfactory), percentage of cells positively stained for E-cadherin (0, 1, 5, 10, 20, 30, 40, 50, 60, 70, 80, 90 or 100%) and the intensity of staining (0 = negative, 1 = weak, 2 = intermediate, and 3 = strong). Reproducibility of IHC scoring was assessed based on evaluation of 200 images, by the two cytotechnologist and a pathologist (M.E.S.); inter- and intra-observer agreement was excellent (weighted kappa ≥90%; P < 0.001). A summary E-cadherin score was calculated using the product of % positive tumor cells and intensity (range of 0–300)^23–25^. For patients with multiple spots, the maximum E-cadherin score across the spots was calculated for analysis. The median and interquartile range for the E-cadherin score did not vary substantially across the 12 studies (Supplementary Table 2). Tumors having a score of <100 were classified as E-cadherin low and those with a score ≥100 as E-cadherin high. Representative images are shown in Supplementary Figure 1. This cut-point was informed by the known relationship between E-cadherin expression and lobular histology supported by evidence in the literature^23,32,33^. Further for sensitivity analysis, we also evaluated a more stringent cut-point defining E-cadherin loss with a score = 0 (i.e. no expression of E-cadherin).

### Assessment of other tumor markers

Assessment of the tumor markers ER, PR and human epidermal growth factor receptor 2 (HER2), and the definition of positive expression of the tumor markers varied across studies. For the majority of cases (N = 1891, 32%), ER status were primarily extracted from medical records, 15% (N = 908) had ER obtained from IHC staining of whole sections and 28% (N = 1685) had ER obtained from IHC staining of TMAs. Previous publications from participating groups in the current study show good concordances between marker status from medical records and standardized measurements from TMA analyses^34–36^.

### Statistical analysis

#### Case-case analyses

As we performed all staining at the NCI to minimize batch effects, a pooled analysis was conducted using data from all 12 studies. We performed case-case analyses to assess whether there was heterogeneity in risk factor associations by E-cadherin breast tumor expression. We used logistic regression models to estimate case-case odds ratios (OR) and 95% confidence intervals (CIs) where E-cadherin low vs. E-cadherin high expression was the outcome and risk factors the explanatory variables. The ORs were interpreted as the risk factor associations of E-cadherin low disease compared to E-cadherin high disease. For each risk factor, the category that has been shown to be associated with the lowest overall breast cancer risk in the literature was selected as the reference category. Thus, the case-case OR >1 can be interpreted to mean that the risk factor examined in the analysis is more strongly associated with E-cadherin low tumors than with E-cadherin high tumors (OR_E-cadherin low vs. control_ > OR_E-cadherin high vs. control_). Heterogeneity by E-cadherin subtype were tested using global F test^37^. Because E-cadherin expression may vary by age and study site^23,30,31^ all models were adjusted for age (in 10-year categories) and study site. Given that ER status is an important marker of etiologic heterogeneity^38^, we stratified all analyses by ER status (ER+, ER−). Among ER-positive tumors, we also evaluated associations after stratification by histology (lobular, ductal/mixed); this was not done among ER-negative tumor due to small numbers. To assess the variation in results by study for risk factors that showed evidence of a differential association by E-cadherin expression, we fitted study*risk factor interaction terms in the models to estimate p-heterogeneity by study using the likelihood ratio test; P < 0.20 was considered suggestive evidence of between-study heterogeneity^37^. In sensitivity analysis, we also assessed associations with risk factors using a more stringent definition of E-cadherin loss, where loss of E-cadherin was defined as those cases with a score of 0.

#### Survival analysis

For survival analysis, we further excluded patients with distant metastases at diagnosis of the primary tumor (N = 63) and those who were missing vital status (N = 174). In total, 5,696 invasive breast cancer cases from 12 BCAC studies were included in the survival analysis. A total of 1,085 deaths were observed within 10 years of diagnosis, 671 due to cancer. We calculated the survival time for each case as the difference between the date of diagnosis and the date of death or censoring. Analyses were left censored for time to study entry to allow for inclusion of prevalent cases. End of follow-up was defined as the date of death, date of last follow-up or 10 years, whichever came first. Hazard ratios (HR) and 95% CIs for all-cause mortality and breast cancer-specific mortality were estimated using Cox regression models, using study site as a stratifying factor. Multivariable Cox models were adjusted for potential confounders: age at diagnosis (in 10-year categories), tumor grade (well/moderately differentiated, poorly differentiated, or unknown), tumor size (≤2, >2 cm, or unknown), node status (positive, negative, or unknown), HER2 status (positive, negative, or unknown), and histology (ductal/mixed, lobular, other, or unknown). To assess whether the associations vary by tumor characteristics, we also estimated HRs and 95% CI by ER status (positive, negative, unknown), HER2 status (positive, negative, unknown), and, among ER-positive tumors, histology (lobular, ductal/mixed, other/unknown).

All statistical tests were two-sided with 5% type-I error. All pooled analyses were performed using the SAS software version 9.3 (SAS Institute, Inc, Cary, NC).

## Results

### Study and tumor characteristics by E-cadherin expression

The median age at breast cancer diagnosis was 52 years with some variation by study. E-cadherin low expression by study ranged from 10% to 31% (Supplementary Table 2).

Table 1 presents the distribution of clinicopathologic features by level of E-cadherin tumor tissue expression (low/high). E-cadherin low tumors were more likely to be lobular, well/moderately differentiated (low grade), larger in size (>2 cm), and HER2-negative compared to E-cadherin high tumors (*P* ≤ 0.005; Table 1). These associations were generally consistent across studies (Supplementary Table 3).

**Table 1:** Distribution of select clinicopathologic features among breast cancer cases by E-cadherin tumor tissue expression levels in the 12 participating BCAC studies.

Characteristics	Pooled Data Set		
	E-cadherin low (Score < 100)	E-cadherin high (Score ≥ 100)	P-value
	(N = 1191)	(N = 4742)	
Histology, n (%)			
Ductal	580 (50.9)	3463 (78.4)	
Lobular	410 (36.0)	385 (8.7)	
Mixed	65 (5.7)	251 (5.7)	
Other	84 (7.4)	317 (7.2)	<0.0001
Grade, n (%)			
Well/Moderately differentiated	790 (72.2)	2846 (65.2)	
Poorly differentiated	304 (27.8)	1522 (34.8)	<0.0001
Tumor size, n (%)			
≤2 cm	581 (53.9)	2500 (58.7)	
>2 cm	497 (46.1)	1761 (41.3)	0.005
Axillary node involvement, n (%)			
Negative	637 (57.5)	2462 (55.9)	
Positive	471 (42.5)	1944 (44.1)	0.33
ER status, n (%)			
Negative	286 (25.8)	1135 (26.2)	
Positive	822 (74.2)	3206 (73.9)	0.82
PR status, n (%)			
Negative	391 (36.5)	1606 (38.8)	
Positive	679 (63.5)	2533 (61.2)	0.18
HER2 status, n (%)			
Negative	740 (86.3)	2640 (78.0)	
Positive	118 (13.8)	744 (22.0)	<0.0001

P-values were estimated using the chi-squared test.

Abbreviations: E-cad, E-cadherin; ER, estrogen receptor; PR, progesterone receptor.

### Case-case analyses of risk factor associations with E-cadherin tissue expression among ER-positive tumors overall and stratified by histology

Table 2 presents risk factor associations for ER-positive breast cancers overall and stratified by histology. Among ER-positive cases, compared with E-cadherin high tumors E-cadherin low tumors were marginally associated with ever use of menopausal hormones compared with never users (OR = 1.24, 95% CI = 0.97–1.59, *P-*het = 0.08). No consistent associations were observed for E-cadherin status by age at menarche, number of live births, age at live birth, or anthropometric measurements (BMI and height; Supplementary Table 4).

**Table 2:** Case-case analyses of established breast cancer risk factors with E-cadherin tumor tissue expression (low/high) stratified by tumor histology among estrogen receptor (ER)-positive tumors.

Risk Factor	ER-positive Tumors				ER-positive Lobular Tumors				ER-positive Ductal/Mixed Tumors			
	No. Studies	Cases, N (E-cadherin Low/High)	OR (95% CI)^a^	P-het^b^	No. Studies	Cases, N (E-cadherin Low/High)	OR (95% CI)^a^	P-het^b^	No. Studies	Cases, N (E-cadherin Low/High)	OR (95% CI)^a^	P-het^b^
Family history of breast cancer	10				9				10			
Absent		486/1935	1.0 (Ref)			203/179	1.0 (Ref)			232/1497	1.0 (Ref)	
Present		236/735	1.18 (0.96–1.46)	0.12		97/79	0.98 (0.64–1.51)	0.93		121/589	1.19 (0.89–1.58)	0.24
Age at menarche	10				8				10			
≤12 years		171/703	0.95 (0.76–1.20)			76/73	0.85 (0.55–1.33)			85/566	0.88 (0.65–1.21)	
13 years		155/497	1.14 (0.91–1.44)			74/47	1.45 (0.91–2.34)			66/406	0.92 (0.66–1.28)	
≥14 years		266/933	1.0 (Ref)	0.33		117/98	1.0 (Ref)	0.11		127/736	1.0 (Ref)	0.72
Parity	10				8				10			
Nulliparous		112/375	0.97 (0.76–1.25)			51/28	1.64 (0.97–2.77)			47/309	0.81 (0.57–1.16)	
1		108/510	0.74 (0.58–0.95)			57/52	1.04 (0.66–1.64)			44/397	0.68 (0.47–0.98)	
≥2		399/1377	1.0 (Ref)	0.06		168/156	1.0 (Ref)	0.18		203/1100	1.0 (Ref)	0.09
Age at first birth (among parous women)	8				7				8			
<20 years		44/137	1.0 (Ref)			15/9	1.0 (Ref)			27/110	1.0 (Ref)	
20–24 years		192/718	0.80 (0.54–1.16)			88/85	0.62 (0.25–1.52)			93/565	0.63 (0.39–1.03)	
25–29 years		150/530	0.79 (0.53–1.17)			66/63	0.56 (0.22–1.42)			73/421	0.61 (0.37–1.01)	
≥30 years		83/316	0.74 (0.48–1.13)	0.57		39/34	0.64 (0.24–1.70)	0.68		39/253	0.54 (0.31–0.95)	0.17
Oral contraceptive use (among women at age ≤ 50 years^c^)	5				4				5			
Never		49/257	1.0 (Ref)			25/23	1.0 (Ref)			22/207	1.0 (Ref)	
Ever		88/320	1.39 (0.87–2.21)	0.16		32/23	1.00 (0.37–2.67)	1.00		52/267	1.51 (0.80–2.84)	0.21
Any menopausal hormone (MH) use (among women at age > 50 years^c^)	7				6				7			
Never		192/721	1.0 (Ref)			92/80	1.0 (Ref)			82/564	1.0 (Ref)	
Ever		182/568	1.24 (0.97–1.59)	0.08		93/72	1.23 (0.78–1.94)	0.38		66/429	0.99 (0.69–1.43)	0.96
Type of ever MH use (among women at age > 50 years^c^)	7				6				7			
Never		192/721	1.0 (Ref)			92/80	1.0 (Ref)			82/564	1.0 (Ref)	
Estrogen only		23/66	1.18 (0.68–2.03)			8/13	0.72 (0.26–2.01)			9/44	0.93 (0.41–2.11)	
Estrogen + Progestin		31/137	1.14 (0.73–1.79)			14/15	0.92 (0.40–2.08)			14/102	1.36 (0.70–2.64)	
Unknown		128/365	1.29 (0.97–1.72)	0.34		71/44	1.53 (0.89–2.62)	0.35		43/283	0.91 (0.59–1.40)	0.76

^a^Logistic regression analyses were used to estimate the associations between E-cadherin tumor tissue expression and established breast cancer risk factors using E-cadherin expression levels (low vs. high) as the outcome variable and the risk factors as the independent variable, adjusted for age (10-year categories) and study site.

^b^P-values for heterogeneity by E-cadherin subtype were estimated using global F test, adjusted for age and study site.

^c^Age ≤ 50 years was used as a proxy for premenopausal status and age >50 years was used as a proxy for postmenopausal status.

Abbreviations: OR = odds ratio, CI = confidence interval, ER = estrogen receptor

Note: For each variable, the category that has been shown to be associated with the lowest overall breast cancer risk in the literature was selected as the reference category. The case-case OR may be interpreted as the ratio of case-control ORs for E-cadherin low tumors (vs. controls) and E-cadherin high tumors (vs. controls). The case-case OR >1 may suggest that the risk factor association is more strongly associated with E-cadherin low tumors than with E-cadherin high tumors (OR_E-cadherin low vs. control_ > OR_E-cadherin high vs. control_).

Among women with ER-positive tumors, we observed a difference by E-cadherin status for number of live births; women who had 1-birth were less likely to have E-cadherin low expression than women with 2 or more births (OR = 0.74, 95% CI = 0.58–0.95, Table 2); however no trend was present, based on the result of nulliparous women. This relationship was driven by the ductal/mixed tumors while in contrast, for lobular tumors nulliparous women had more frequent loss of E-cadherin compared to those with two or more live births. Other breast cancer risk factors examined, family history of breast cancer, age at menarche, age at menopause, age at first birth, OC and menopausal hormone use, did not exhibit heterogeneity by E-cadherin expression.

Among ER-positive breast cancers of ductal histology, no breast cancer risk factors examined exhibited heterogeneity in their associations by E-cadherin expression (Table 2 and Supplementary Table 4). Analyses using a score of 0 to define E-cadherin loss are presented in Supplemental Table 5–6. In these sensitivity analysis, we observed a stronger relationship with E-cadherin loss with ER expression (Supplemental Table 5), and analysis by risk factors (Supplemental Table 6) showed ever use of menopausal hormones more likely to have E-cadherin loss (defined as score = 0) compared to never users among ER-positive tumors (OR = 1.57, 95% CI = 1.06–2.33, p = 0.02), other factors did not show significant differences.

### Case-case analyses of risk factor associations with E-cadherin tissue expression among ER-negative tumors

We did not find differences by E-cadherin expression among ER-negative breast cancers (Table 3), although the result for OC use was marginal. Cases that reported ever use of OC’s were more likely to be E-cadherin low compared with E-cadherin high tumors (OR = 1.97, 95% CI = 0.96–4.06, *P*-het = 0.06). No significant associations for E-cadherin status were observed for anthropometric measurements including BMI and height (Supplementary Table 4). There were too few cases to evaluate with this more stringent cut-point of 0 to define E-cadherin loss for ER-negative cases.

**Table 3:** Case-case analyses of established breast cancer risk factors with E-cadherin tumor tissue expression (low/high) among ER-negative tumors.

Risk Factor	Estrogen Receptor Negative Tumors			
	No. of Cases	Cases, N (E-cadherin Low/High)	OR (95% CI)^a^	P-het^b^
Family history of breast cancer	9			
Absent		173/660	1.0 (Ref)	
Present		60/207	1.32 (0.88–1.97)	0.18
Age at menarche	8			
≤12 years		69/225	1.27 (0.87–1.85)	
13 years		43/182	0.94 (0.62–1.44)	
≥14 years		81/291	1.0 (Ref)	0.34
Parity	9			
Nulliparous		27/108	0.82 (0.51–1.33)	
1		48/190	0.82 (0.56–1.21)	
≥2		126/441	1.0 (Ref)	0.52
Age at first birth (among parous women)	6			
<20 years		18/59	1.0 (Ref)	
20–24 years		64/257	0.84 (0.46–1.54)	
25–29 years		53/144	1.26 (0.67–2.35)	
≥30 years		22/86	0.93 (0.45–1.93)	0.32
Oral contraceptive use (among women at age ≤50 yearsc)	5			
Never		23/125	1.0 (Ref)	
Ever		37/132	1.97 (0.96–4.06)	0.06
Menopausal hormone (MH) use (among women at age >50 yearsc)	6			
Never		63/207	1.0 (Ref)	
Ever		48/154	1.25 (0.78–2.00)	0.35
Type of ever MH use (among women at age >50 yearsc)	6			
Never		63/207	1.0 (Ref)	
Estrogen only		4/27	0.46 (0.14–1.45)	
Estrogen + Progestin		14/32	1.67 (0.80–3.49)	
Unknown		30/95	1.38 (0.78–2.46)	0.16

^a^Logistic regression analyses were used to estimate the associations between E-cadherin tumor tissue expression and established breast cancer risk factors using E-cadherin expression levels (low vs. high) as the outcome variable and the risk factors as the independent variable, adjusted for age (10-year categories) and study site.

^b^P-values for heterogeneity by E-cadherin subtype were estimated using global F test, adjusted for age and study site.

^c^Age ≤ 50 years was used as a proxy for premenopausal status and age > 50 years was used as a proxy for postmenopausal status.

Abbreviations: OR = odds ratio, CI = confidence interval, ER = estrogen receptor

Note: For each variable, the category that has been shown to be associated with the lowest overall breast cancer risk in the literature was selected as the reference category. The case-case OR may be interpreted as the ratio of case-control ORs for E-cadherin low tumors (vs. controls) and E-cadherin high tumors (vs. controls). The case-case OR > 1 may suggest that the risk factor association is more strongly associated with E-cadherin low tumors than with E-cadherin high tumors (OR_E-cadherin low vs. control_ > OR_E-cadherin high vs. control_).

### E-cadherin expression and survival by tumor subtypes

The mean follow-up time was 9.6 years and results for all-cause and breast cancer specific survival are presented in Table 4. E-cadherin expression showed no significant associations with survival in multivariable models overall, or in any of the tumor subtypes.

**Table 4:** 10-year Hazard ratios (HR) and 95% confidence intervals (CI) for all-cause and breast cancer specific mortality according to E-cadherin tumor expression (low/high): a pooled analysis of 12 participating Breast Cancer Association Consortium studies.

	N	Overall Deaths	All-cause mortality		Breast cancer Deaths	Breast cancer-specific mortality	
			Age-adjusted HR	Multivariable adjusted HR		Age-adjusted HR	Multivariable adjusted HR
			(95% CI)^a^	(95% CI)^b^		(95% CI)^a^	(95% CI)^b^
**By ER status**							
ER+	3952	668	0.97 (0.81–1.17)	0.98 (0.80–1.18)	384	1.00 (0.78–1.28)	1.02 (0.79–1.31)
ER−	1380	376	1.14 (0.88–1.47)	1.02 (0.78–1.33)	258	0.99 (0.73–1.34)	0.86 (0.62–1.18)
**By histology among ER + tumors**							
Lobular	630	63	0.84 (0.58–1.21)	0.83 (0.57–1.22)	67	0.85 (0.52–1.40)	0.84 (0.50–1.39)
Ductal/mixed	2879	289	1.05 (0.82–1.35)	1.03 (0.80–1.32)	300	1.11 (0.80–1.52)	1.12 (0.81–1.55)
					17		
**By HER2**							
HER2+	839	223	0.68 (0.48–0.96)	0.74 (0.51–1.06)	156	0.68 (0.45–1.02)	0.66 (0.43–1.02)
HER2−	3318	635	1.10 (0.91–1.32)	1.11 (0.91–1.36)	390	1.11 (0.86–1.42)	1.12 (0.86–1.44)

^a^Hazard ratios and 95% confidence intervals were estimated using stratified Cox models (stratified by study site), adjusted for age at diagnosis (in 10-year categories).

^b^For analyses by ER status, hazard ratios and 95% confidence intervals were estimated using stratified Cox models (stratified by study site), adjusted for age at diagnosis (in 10-year categories), tumor grade (well/moderately differentiated, poorly differentiated, or unknown), tumor size (≤2, >2 cm, or unknown), axillary node involvement (negative, positive, unknown), histology (ductal/mixed, lobular, other/unknown), and HER2 status (positive, negative, or unknown). For analyses by histology among ER + tumors, hazard ratios and 95% confidence intervals were estimated using stratified Cox models (stratified by study site), adjusted for age at diagnosis (in 10-year categories), tumor grade (well/moderately differentiated, poorly differentiated, or unknown), tumor size (≤2, >2 cm, or unknown), axillary node involvement (negative, positive, unknown), and HER2 status (positive, negative, or unknown). For analyses by HER2 status, hazard ratios and 95% confidence intervals were estimated using stratified Cox models (stratified by study site), adjusted for age at diagnosis (in 10-year categories), tumor grade (well/moderately differentiated, poorly differentiated, or unknown), tumor size (≤2, >2 cm, or unknown), axillary node involvement (negative, positive, unknown), histology (ductal/mixed, lobular, other/unknown), and ER status (positive, negative, unknown).

## Discussion

In our study of nearly 6,000 breast cancer patients, with centrally stained and scored TMA slides, our analyses demonstrated E-cadherin loss was significantly associated with lobular histology consistent with previous work. We found limited evidence of heterogeneity of E-cadherin loss, except for menopausal hormone use, to vary by risk factors or with 10-year breast cancer specific survival within tumor subtypes.

Analysis by tumor characteristics and E-cadherin loss showed significant associations with lobular histology, low grade, larger tumor size and lack of HER2 staining, consistent with previous studies^39^. Lobular breast cancers feature noncohesive cells that are individually dispersed or arranged in a single file pattern, a phenotype that has been attributed to dysregulation of cell-cell adhesion, primarily by loss of E-cadherin protein expression^40,41^. Lobular breast cancers because of their single file pattern tend to be harder to detect in screening and hence, are larger when diagnosed^39^, consistent with our data showing E-cadherin loss associated with larger tumor size.

We also observed a relationship between low E-cadherin expression and ever use of menopausal hormones among ER-positive tumors, which was more pronounced when we used a more stringent cut-point of E-cadherin loss (with a score of 0). Numerous studies have shown that menopausal hormone use, particularly combined estrogen-progestin therapy, to be more strongly associated with lobular tumors than with ductal tumors and that reduced use of menopausal hormones is associated with a declining incidence rate of lobular cancers at the population level^11–13,15–17,19–22,42,43^. Further, we also observed among ER-negative breast cancers use of OCs compared to never users to be almost twice as likely to have E-cadherin loss, although not statistically significant. Given that findings suggesting that the relationship between menopausal hormones or OC and breast cancer risk are strongly influenced by recent exposure, it is possible that a true association in our data was attenuated by our reliance on ever as opposed to current use. Given prior epidemiologic studies and *in vitro* data showing that estrogen may lower E-cadherin expression, the observed relationship with OC or menopausal hormone use may be plausible^44^.

From our analysis of breast cancer risk factors among ER-positive tumors we observed that women who had one birth were less frequently E-cadherin low compared to women who had two or more live births. We saw an opposite relationship among lobular tumors where nulliparous women had more frequent loss of E-cadherin compared to those with two or more live births. Whether E-cadherin loss in tumors is related to reproductive characteristics requires larger datasets. With regards to genetic factors, mutation profiling studies targeted at *CDH1* suggest mutations to be rare and unlikely to explain loss (33/507 based on TCGA data)^45,46^.

We did not observe associations between E-cadherin and breast cancer specific survival in multivariable models as reported in previous studies^24–28^. Our data, based on the largest analysis of its kind, do not support E-cadherin as an important marker of survival in breast cancer patients.

Strengths of our study include the use of large, pooled analysis, centrally stained and scored E-cadherin data which allowed for the reduction of any systematic bias that may have been introduced across participating studies. Limitations of this study include limited power for analysis of tumor subgroups due to smaller numbers especially for survival analysis. Although staining of E-cadherin was performed centrally, we had lower than expected percentages of E-cadherin associated lobular breast cancers, which may reflect variation in calling of histologic subtypes, but could also indicate the need for molecular profiling methods or more detailed image analysis studies on the compartment of where E-cadherin is stained needed, for defining E-cadherin loss^47,48^. In fact, the percentage of E-cadherin low lobular carcinomas varied by study, which could reflect tissue factors that influenced staining, variability in classification of cancers as lobular including potential sampling issues if TMA’s did not capture fully lobular morphology, or factors related to populations and relative frequency of risk exposures.

In summary, this study provides limited evidence for heterogeneity in risk factor associations or for differences in survival by E-cadherin tumor tissue expression. Our data are consistent with molecular profiling studies showing distinctive expression of genes associated with E-cadherin signaling among ER-positive ductal and lobular carcinomas^10,49^. Evaluating genetic susceptibility markers and E-cadherin loss, where data suggest that genetic susceptibility factors may influence loss of E-cadherin expression, might provide new insights on pathways of E-cadherin loss consistent with histology analysis^23,50^. Future studies using comprehensive molecular subtyping data, including histology, hormone markers and mRNA, might provide new insights on common and distinct molecular pathways of E-cadherin loss as well as tumor heterogeneity^51^.

## Declarations

### Ethics approval and consent to participate

Study participants were recruited under protocols approved by the Institutional Review Board at each institution, and all subjects provided informed consent or did not opt-out, depending on national regulations.

Amsterdam Breast Cancer Study (ABCS), Netherlands Leiden University Medical Center (LUMC) Commissie Medische Ethiek and Protocol Toetsingscommissie van het Nederlands Kanker Instituut/Antoni van Leeuwenhoek Ziekenhuis; Spanish National Cancer Centre Breast Cancer Study (CNIO-BCS) Spain Hospital Universitario La Paz Comite Etico de Investigacion Clinica; ESTHER Breast Cancer Study (ESTHER) GermanyRuprecht-Karls-Universitat Medizinische Fakultat Heidelberg Ethikkommission; Helsinki Breast Cancer Study (HEBCS) Finland Helsingin ja uudenmaan sairaanhoitopiiri (Helsinki University Central Hospital Ethics Committee); Kuopio Breast Cancer Project (KBCP) Finland Pohjois-Savon Sairraanhoitopiirin Kuntayhtyma Tutkimuseettinen Toimikunta; Kathleen Cuningham Foundation Consortium for Familial Breast Cancer/Australian Ovarian Cancer Study (kConFab/AOCS) Australia kConFab: The Queenland Institute of Medical Research Human Research Ethics Committee (QIMR-HREC) AOCS: Peter MacCallum Cancer Centre Ethics Committee; Mayo Clinic Breast Cancer StudY (MCBCS) USA Mayo Clinic IRB; Leiden University Medical Centre Breast Cancer Study (ORIGO) Netherlands Medical Ethical Committee and Board of Directors of the Leiden University Medical Center (LUMC); NCI Polish Breast Cancer Study (PBCS) Poland National Institute of Health (NIH) IRB; Prospective Study of Outcomes in Sporadic Versus Hereditary Breast Cancer (POSH) UK South West Multi-centre Research Ethics Committee; Rotterdam Breast Cancer Study (RBCS) Netherlands Medische Ethische Toetsings Commissie Erasmus Medisch Centrum; UK Breakthrough Generations Study (UKBGS) UK South East Multi-Centre Research Ethics Committee.

### Availability of data and materials

The datasets generated and/or analyzed during the current study are not publicly available due to privacy and ethical approvals but are available from the corresponding author on reasonable request.

## Electronic supplementary material

Supplementary material
