# Supplementary material for: E-cadherin breast tumor expression, risk factors and survival: Pooled analysis of 5,933 cases from 12 studies in the Breast Cancer Association Consortium
Source: Sci Rep. 2018 Apr 26;8:6574. doi: 10.1038/s41598-018-23733-4 (PMC5920115; doi:10.1038/s41598-018-23733-4)
Supplement: Supplementary file 1 — Supplementary material [file 41598_2018_23733_MOESM1_ESM.docx]

**SUPPLEMENTARY MATERIAL**

**E-cadherin breast tumor expression, risk factors and survival: Pooled analysis of 5,933 cases from 12 studies in the Breast Cancer Association Consortium**

Hisani N. Horne^1,2^*^#^, Hannah Oh^1,3^*^#^, Mark E. Sherman^4#^, Maya Palakal^1^, Stephen M. Hewitt^5^, Marjanka K. Schmidt^6,7^, Roger L. Milne^8,9^, David Hardisson, Javier Benitez^10,11^, Carl Blomqvist^12^, Manjeet K. Bolla^13^, Hermann Brenner^14-16^, Jenny Chang-Claude^17,18^, Renata Cora^19^, Fergus J. Couch^20^, Katarina Cuk^14^, Peter Devilee^21,22^, Douglas F. Easton^13,23^, Diana M. Eccles^24^, Ursula Eilber^17^, Jaana M. Hartikainen^25-27^, Päivi Heikkilä^28^, Bernd Holleczek^29^, Maartje J. Hooning^30^, Michael Jones^31^, Renske Keeman^6^, Arto Mannermaa^25-27^, John W.M. Martens^30^, Taru A. Muranen^32^, Heli Nevanlinna^32^, Janet E. Olson^33^, Nick Orr^34^, Jose I.A. Perez^35^, Paul D.P. Pharoah^13,23^, Kathryn J. Ruddy^33^, Kai-Uwe Saum^14^, Minouk J. Schoemaker^31^, Caroline Seynaeve^30^, Reijo Sironen^25-27^, Vincent T.H.B.M. Smit^21^, Anthony J. Swerdlow^31,36^, Maria Tengström^25,37,38^, Abigail S. Thomas^33^, A. Mieke Timmermans^30^, Rob A.E.M. Tollenaar^39^, Melissa A. Troester^40^, Christi J. van Asperen^41^, Carolien H.M. van Deurzen^42^, Flora F. Van Leeuwen^6^, Laura J. Van't Veer^6^, Montserrat García-Closas^1#^, Jonine D. Figueroa^1,43#^

*These authors contributed equally to this work.

^#^These authors were part of the writing group.

1. Division of Cancer Epidemiology and Genetics, National Cancer Institute, Rockville, MD, USA.

2. Division of Molecular Genetics & Pathology, US Food and Drug Administration, Silver Spring, MD, USA.

3. Department of Medicine, Section of Population Sciences, Rutgers Cancer Institute of New Jersey, New Brunswick, NJ, USA.

4. Health Sciences Research, Mayo Clinic, Jacksonville,FL, USA.

5. Center for Cancer Research, National Cancer Institute, Bethesda, MD, USA.

6. Division of Molecular Pathology, The Netherlands Cancer Institute - Antoni van Leeuwenhoek Hospital, Amsterdam, The Netherlands.

7. Division of Psychosocial Research and Epidemiology, The Netherlands Cancer Institute - Antoni van Leeuwenhoek hospital, Amsterdam, The Netherlands.

8. Cancer Epidemiology & Intelligence Division, Cancer Council Victoria, Melbourne, Victoria, Australia.

9. Centre for Epidemiology and Biostatistics, Melbourne School of Population and Global health, The University of Melbourne, Melbourne, Victoria, Australia.

10. Human Cancer Genetics Program, Spanish National Cancer Research Centre, Madrid, Spain.

11. Centro de Investigación en Red de Enfermedades Raras (CIBERER), Valencia, Spain.

12. Department of Oncology, Helsinki University Hospital, University of Helsinki, Helsinki, Finland.

13. Centre for Cancer Genetic Epidemiology, Department of Public Health and Primary Care, University of Cambridge, Cambridge, UK.

14. Division of Clinical Epidemiology and Aging Research, German Cancer Research Center (DKFZ), Heidelberg, Germany.

15. Division of Preventive Oncology, German Cancer Research Center (DKFZ) and National Center for Tumor Diseases (NCT), Heidelberg, Germany.

16. German Cancer Consortium (DKTK), German Cancer Research Center (DKFZ), Heidelberg, Germany.

17. Division of Cancer Epidemiology, German Cancer Research Center (DKFZ), Heidelberg, Germany.

18. Research Group Genetic Cancer Epidemiology, University Cancer Center Hamburg (UCCH), University Medical Center Hamburg-Eppendorf, Hamburg, Germany.

19. Independent contractor, CT(ASCP), MB (ASCP), National Cancer Institute, Bethesda, MD, USA.

20. Department of Laboratory Medicine and Pathology, Mayo Clinic, Rochester, MN, USA.

21. Department of Pathology, Leiden University Medical Center, Leiden, The Netherlands.

22. Department of Human Genetics, Leiden University Medical Center, Leiden, The Netherlands.

23. Centre for Cancer Genetic Epidemiology, Department of Oncology, University of Cambridge, Cambridge, UK.

24. Cancer Sciences Academic Unit, Faculty of Medicine, University of Southampton, Southampton, UK.

25. Translational Cancer Research Area, University of Eastern Finland, Kuopio, Finland.

26. Institute of Clinical Medicine, Pathology and Forensic Medicine, University of Eastern Finland, Kuopio, Finland.

27. Imaging Center, Department of Clinical Pathology, Kuopio University Hospital, Kuopio, Finland.

28. Department of Pathology, Helsinki University Hospital, University of Helsinki, Helsinki, Finland.

29. Saarland Cancer Registry, Saarbrücken, Germany.

30. Department of Medical Oncology, Family Cancer Clinic, Erasmus MC Cancer Institute, Rotterdam, The Netherlands.

31. Division of Genetics and Epidemiology, The Institute of Cancer Research, London, UK.

32. Department of Obstetrics and Gynecology, Helsinki University Hospital, University of Helsinki, Helsinki, Finland.

33. Department of Health Sciences Research, Mayo Clinic, Rochester, MN, USA.

34. The Breast Cancer Now Toby Robins Research Centre, The Institute of Cancer Research, London, UK.

35. Servicio de Cirugía General y Especialidades, Hospital Monte Naranco, Oviedo, Spain.

36. Division of Breast Cancer Research, The Institute of Cancer Research, London, UK.

37. Cancer Center, Kuopio University Hospital, Kuopio, Finland.

38. Institute of Clinical Medicine, Oncology, University of Eastern Finland, Kuopio, Finland.

39. Department of Surgery, Leiden University Medical Center, Leiden, The Netherlands.

40. Department of Pathology and Laboratory Medicin, Gillings School of Global Public Health, Lineberger Comprehensive Cancer Center, University of North Carolina, Chapel Hill, NC, USA.

41. Department of Clinical Genetics, Leiden University Medical Center, Leiden, The Netherlands.

42. Department of Pathology, Erasmus University Medical Center, Rotterdam, The Netherlands.

43. Usher Institute of Population Health Sciences and Informatics, The University of Edinburgh Medical School, Edinburgh, UK.

**TABLES & FIGURES**

**Supplemental Figure 1: E-cadherin staining in tumor microarrays**

**Supplemental Figure 1 Legend:** Representative immunohistochemical (IHC) staining of invasive breast tumors with E-cadherin low (A) and high (B) tissue expression.

| **Supplementary Table 1.** Description of study design and case contributions for the 12 studies participating the Breast Cancer Consortium (BCAC) included in the E-cadherin TMA study | | | | | | | |  |
| --- | --- | --- | --- | --- | --- | --- | --- | --- |
| **Study Name [Reference]** | **Country** | **Study Design** | **Definitions of case patients and control subjects** | **Age at diagnosis, Mean (SD)** | **Invasive Cases,**  **N** | **Cases on TMA,**  **N** | **Invasive Cases with E-cadherin data,**  **N (%)** |  |
| Amsterdam Breast Cancer Study (ABCS) [1] | Netherlands | Mixed | Case patients aged <50 and diagnosed from 1974-1994 in 4 Dutch hospitals. | 42.6 (5.1) | 3585 | 756 | 731 (96.7) |  |
| Spanish National Cancer Centre Breast Cancer Study (CNIO-BCS) [2] | Spain | Mixed | Two groups of cases from, 1) 574 consecutive breast cancer cases unselected for family history from 3 public hospitals and 2) 291 cases with at least one first degree relative also affected with breast cancer (2000-2005). Controls are women attending the Menopause Research Center between 2000-2005 and female members of the College of Lawyers. | 60.3 (8.1) | 1023 | 165 | 126 (76.4) |  |
| ESTHER Breast Cancer Study (ESTHER) [3] | Germany | Population-based case-control | Breast cancer cases in all hospitals of the state of Saarland, from 2001-2003. Controls are a random sample of women undergoing a routine health check-up in Saarland, 2000-2002. | 62.0 (6.6) | 496 | 264 | 259 (98.1) |  |
| Helsinki Breast Cancer Study (HEBCS) [4] | Finland | Mixed | Consecutive case patients from 1)Dept. of Oncology (1997-8 & 2000), 2) the Dept. of Surgery (2001-2004), and 3) familial breast cancer patients from the Depts. of Oncology and Clinical Genetics (1995- ). All from Helsinki University Central Hospital. Controls are healthy females from the same geographical region in Southern Finland in 2003. | 55.5 (12.5) | 2263 | 1095 | 1029 (94.0) |  |
| Kuopio Breast Cancer Project (KBCP) [5] | Finland | Hospital-based prospective clinical cohort | Women seen at Kuopio University Hospital between 1990 and 1995 for breast symptoms and were found to have breast cancer. Controls are age and long-term area-of-residence matched subjects selected from the National Population Register between 1990-1995. | 59.3 (14.8) | 459 | 388 | 372 (95.9) |  |
| Kathleen Cuningham Foundation Consortium for Research into Familial Breast Cancer / Australian Ovarian Cancer Study (kConFab/AOCS) [6] | Australia | Mixed | Case patients from multiple-case breast and breast-ovarian families recruited through cancer clinics. Negative for BRCA1 and BRCA2 mutations included in BCAC. Controls are selected from the Australian Ovarian Cancer Study. | 45.1 (9.6) | 522 | 363 | 337 (92.8) |  |
| Mayo Clinic Breast Cancer Study (MCBCS) [7] | U.S.A. | Clinic-based case-control | Incident cases residing in 6 (MN, WI, IA, IL, ND, SD) US states, seen at the mayo Clinic in Rochester, MN from 2002-210. Controls are women without cancer presenting for general medical examination at the Mayo Clinic from 2002-2010. | 58.1 (13.0) | 1796 | 524 | 454 (86.6) |  |
| Leiden University Medical Center Breast Cancer Study (ORIGO) [8,9] | Netherlands | Mixed | Consecutive case patients diagnosed between 1996 and 2006 in 2 hospitals of South-West Netherlands. No selection of family history. Controls are three groups of subjects: 1) blood bank health donors (1996, 2000 or 2007), 2) people who married a person with high breast cancer risk (1990-1996), and 3) females tested at the local clinical genetics department for familial disease, excluding familial cancer syndromes (1995-2007). | 53.1 (11.8) | 1345 | 475 | 434 (91.4) |  |
| NCI Polish Breast Cancer Study (PBCS) [10] | Poland | Population-based case-control | Incident case patients from 2000-2003 identified through a rapid identification system in participating hospitals covering about 90% of all eligible case patients. Controls are women randomly selected from population lists of all residents of Poland (2000-2003). | 56.0 (9.9) | 2008 | 1332 | 1268 (95.2) |  |
| Prospective Study of Outcomes in Sporadic Versus Hereditary Breast Cancer (POSH) [11, 12] | U.K. | Population-based case-control | Case patients aged 40 or younger at breast cancer diagnosis, recruited across the UK and diagnosed between January 2000 and December 2007. There were no in-house control subjects. | 36.3 (3.5) | 1236 | 631 | 588 (93.2) |  |
| Rotterdam Breast Cancer Study (RBCS) [13] | Netherlands | Hospital-based case-control | Familial breast cancer patients selected from the clinical genetics center at Erasmus Medical Center between 1994-2005. Controls are spouses of mutation-negative siblings of heterozygous Cystic Fibrosis mutation carriers selected from the clinical genetics centre at Erasmus Medical Center between 1996-2006. | 44.7 (10.4) | 722 | 266 | 258 (97.0) |  |
| Generations Study (UKBGS) [14] | U.K. | Cohort | Cohort members who developed breast cancer or in situ disease after entry into the Generations Study (cohort of >100,000 women followed up for breast cancer, recruited during 2003-2010). Controls are women who had not had breast cancer selected 1:1 matching to cases on date of birth, year of entry into the study (2003-2010). | 56.2 (10.7) | 2700 | 95 | 77 (81.1) |  |
| **References: Supplementary Table 1** | | | | | | | | |
| 1. Schmidt,M.K. et al. Breast cancer survival and tumor characteristics in premenopausal women carrying the CHEK2*1100delC germline mutation. J Clin Oncol 25, 64-69 (2007). | | | | | | | | |
| 2. Milne,R.L. et al. ERCC4 associated with breast cancer risk: a two-stage case-control study using high-throughput genotyping. Cancer Res 66, 9420-9427 (2006). | | | | | | | | |
| 3. Widschwendter,M. et al. Epigenotyping in peripheral blood cell DNA and breast cancer risk: a proof of principle study. PLoS One 3, e2656 (2008). | | | | | | | | |
| 4. Heikkinen ,T. et al. The breast cancer susceptibility mutation PALB2 1592delT is associated with an aggressive tumor phenotype. Clin Cancer Res 2009;15:3214-22. | | | | | | | | |
| 5. Hartikainen,J.M. et al. An autosome-wide scan for linkage disequilibrium-based association in sporadic breast cancer cases in eastern Finland: three candidate regions found. Cancer Epidemiol Biomarkers Prev 14, 75-80 (2005). | | | | | | | | |
| 6. Beesley,J. et al. Association between single-nucleotide polymorphisms in hormone metabolism and DNA repair genes and epithelial ovarian cancer: results from two Australian studies and an additional validation set. Cancer Epidemiol Biomarkers Prev 16, 2557-2565 (2007). | | | | | | | | |
| 7. Olson,J.E. et al. A comprehensive examination of CYP19 variation and breast density. Cancer Epidemiol Biomarkers Prev 16, 623-625 (2007). | | | | | | | | |
| 8. de Bock,G.H. et al. Tumour characteristics and prognosis of breast cancer patients carrying the germline CHEK2*1100delC variant. J Med Genet 41, 731-735 (2004). | | | | | | | | |
| 9. Huijts,P.E. et al. Clinical correlates of low-risk variants in FGFR2, TNRC9, MAP3K1, LSP1 and 8q24 in a Dutch cohort of incident breast cancer cases. Breast Cancer Res 9, R78 (2007). | | | | | | | | |
| 10. Garcia-Closas,M. et al. Established breast cancer risk factors by clinically important tumour characteristics. Br J Cancer 95, 123-129 (2006). | | | | | | | | |
| 11. Eccles D, Gerty S, Simmonds P, et al. Prospective study of Outcomes in Sporadic versus Hereditary breast cancer (POSH): study protocol. BMC Cancer 2007;7:160. | | | | | | | | |
| 12. Tapper W, Hammond V, Gerty S, et al. The influence of genetic variation in 30 selected genes on the clinical characteristics of early onset breast cancer. Breast Cancer Res 2008;10(6):R108. | | | | | | | | |
| 13. Hofman,A. et al. The Rotterdam Study: 2010 objectives and design update. Eur J Epidemiol 24, 553-572 (2009). | | | | | | | | |
| 14. Swerdlow,A.J. et al. The Breakthrough Generations Study: design of a long-term UK cohort study to investigate breast cancer aetiology. Br J Cancer 105, 911-917 (2011). | | | | | | | | |

**Supplementary Table 2.** Summary of E-cadherin tumor tissue staining among invasive cases

| **Study Acronym** | **Cases with E-cadherin data,**  **N** | **E-cadherin Score^a^, Median (IQR)** | **E-cadherin Low^b^,**  **N (%)** | **E-cadherin High^b^,**  **N (%)** |
| --- | --- | --- | --- | --- |
| **ABCS** | 731 | 200 (90) | 112 (15.3) | 619 (84.7) |
| **CNIO-BCS** | 126 | 200 (140) | 21 (16.7) | 105 (83.3) |
| **ESTHER** | 259 | 270 (120) | 39 (15.1) | 220 (84.9) |
| **HEBCS** | 1,029 | 180 (120) | 268 (26.0) | 761 (74.0) |
| **KBCP** | 372 | 180 (90) | 116 (31.2) | 256 (68.8) |
| **kConFab/AOCS** | 337 | 200 (100) | 64 (19.0) | 273 (81.0) |
| **MCBCS** | 454 | 270 (90) | 67 (14.8) | 387 (85.2) |
| **ORIGO** | 434 | 180 (160) | 121 (27.9) | 313 (72.1) |
| **PBCS** | 1,268 | 240 (110) | 252 (19.9) | 1,016 (80.1) |
| **POSH** | 588 | 180 (90) | 96 (16.3) | 492 (83.7) |
| **RBCS** | 258 | 255 (120) | 27 (10.5) | 231 (89.5) |
| **UKBGS** | 77 | 200 (90) | 8 (10.4) | 69 (89.6) |

^a^E-cadherin Score = Percent tumor cells staining positive (0-100%) x Staining Intensity (1-3); Range 0-300

^b^E-cadherin low, E-cadherin Score <100 Score; E-cadherin high, E-cadherin score 100-300

Abbreviations: IQR = interquartile range; ABCS= Amsterdam Breast Cancer Study; CNIO-BCS=Spanish National

Cancer Centre Breast Cancer Study; ESTHER= ESTHER Breast Cancer Study; HEBCS=Helsinki Breast Cancer

Study; KBCP= Kuopio Breast Cancer Project; kConFab/AOCS=Kathleen Cuningham Foundation Consortium for

Research into Familial Breast Cancer / Australian Ovarian Cancer Study; MCBCS=Mayo Clinic Breast Cancer

Study; ORIGO=Leiden University Medical Center Breast Cancer Study; PBCS=NCI Polish Breast Cancer Study;

POSH=Prospective Study of Outcomes in Sporadic Versus Hereditary Breast Cancer; RBCS=Rotterdam Breast

Cancer Study; UKBGS=Breakthrough Generations Study.

**Supplementary Table 3. Distribution of select clinicopathologic features among breast cancer cases by E-cadherin by study (See attached excel file)**

| **Supplementary Table 4.** Case-only analyses of anthropometric measures with E-cadherin tumor tissue expression (low/high) stratified by estrogen receptor status and histology | | | | | | | | | |
| --- | --- | --- | --- | --- | --- | --- | --- | --- | --- |
| Risk Factor | Cases, N | OR (95% CI)^a^ | P-het^b^ | P-het |  | Cases, N | OR (95% CI)^a^ | P-het^b^ | P-het |
|  | (E-cadherin Low/High) |  |  | adj for histology^c^ |  | (E-cadherin Low/High) |  |  | adj for histology^c^ |
|  | Estrogen Receptor Positive Tumors | | | |  | Estrogen Receptor Negative Tumors | | | |
| **BMI among women at age ≤50 years**^d^ | | | | | | | | | |
| per 1 kg/m^2^ decrease | 230/956 | 1.01 (0.98-1.04) | 0.55 | 0.72 |  | 99/382 | 0.97 (0.93-1.02) | 0.20 | 0.18 |
| **Body mass index among women at age >50 years**^d^ | | | | | | | | | |
| per 1 kg/m^2^ increase | 408/1388 | 1.01 (0.99-1.03) | 0.46 | 0.40 |  | 122/396 | 0.99 (0.95-1.03) | 0.71 | 0.80 |
| **Body mass index** | | | | | | | | | |
| per 1 kg/m^2^ increase | 640/2361 | 1.00 (0.99-1.02) | 0.69 | 0.59 |  | 221/779 | 1.01 (0.98-1.04) | 0.43 | 0.33 |
| **Height** |  |  |  |  |  |  |  |  |  |
| per 1 cm increase | 557/2104 | 1.00 (0.98-1.01) | 0.70 | 0.98 |  | 199/694 | 1.01 (0.98-1.03) | 0.84 | 0.94 |
|  | Lobular Tumors | | | |  | Ductal/Mixed Tumors | | | |
| **BMI among women at age ≤50 years**^d^ | | | | | | | | | |
| per 1 kg/m^2^ decrease | 78/66 | 0.97 (0.89-1.05) | 0.46 | ~ |  | 132/747 | 1.02 (0.98-1.07) | 0.30 | ~ |
| **Body mass index among women at age >50 years**^d^ | | | | | | | | | |
| per 1 kg/m^2^ increase | 194/166 | 1.02 (0.97-1.06) | 0.48 | ~ |  | 175/1083 | 1.01 (0.97-1.04) | 0.78 | ~ |
| **Body mass index** | | | | | | | | | |
| per 1 kg/m^2^ increase | 273/232 | 1.02 (0.98-1.06) | 0.44 | ~ |  | 308/1846 | 1.00 (0.97-1.02) | 0.79 | ~ |
| **Height** |  |  |  |  |  |  |  |  |  |
| per 1 cm increase | 256/215 | 0.99 (0.96-1.02) | 0.35 | ~ |  | 254/1685 | 1.01 (0.99-1.03) | 0.33 | ~ |
| ^a^Logistic regression analyses were used to estimate the associations between E-cadherin tumor tissue expression and established breast cancer risk factors using E-cadherin expression levels (low vs. high) as the outcome variable and the risk factors as the independent variable, adjusted for age (10-year categories) and study site. | | | | | | | | | |
| ^b^P-values for heterogeneity by E-cadherin subtype were estimated using global F test, adjusted for age and study. | | | | | | | |  |  |
| ^c^P-values for heterogeneity by E-cadherin subtype were estimated using global F test, adjusted for age, study, and tumor histology (Ductal/mixed, lobular, other, unknown). | | | | | | | | |  |
| ^d^Age ≤50 years was used as a proxy for premenopausal status and age >50 years was used as a proxy for postmenopausal status. | | | | | | | | |  |
| Abbreviations: OR=odds ratio, CI=confidence interval, ER=estrogen receptor | | | | |  |  |  |  |  |
| **Note:** For each variable, the category that has been shown to be associated with the lowest overall breast cancer risk in the literature was selected as the reference category. The case-case OR may be interpreted as the ratio of case-control ORs for E-cadherin low tumors (vs. controls) and E-cadherin high tumors (vs. controls). The case-case OR >1 may suggest that the risk factor association is more strongly associated with E-cadherin low tumors than with E-cadherin high tumors (OR_E-cadherin low vs. control_ > OR_E-cadherin high vs. control_). | | | | | | | | | |

| **Supplemental Table 5.** Distribution of select clinicopathologic features among breast cancer cases by E-cadherin tumor tissue expression levels in the 12 participating BCAC studies where E-cadherin low was defined as score=0 and E-cadherin high defined as >0. | | | |
| --- | --- | --- | --- |
|  | Pooled Data Set | | |
| Characteristics | E-cadherin low | E-cadherin high | P-value |
|  | (N=323) | (N=5610) |  |
| Histology, n (%) |  |  |  |
| Ductal | 98 (31.0) | 3945 (75.3) |  |
| Lobular | 189 (59.8) | 606 (11.6) |  |
| Mixed | 22 (7.0) | 294 (5.6) |  |
| Other | 7 (2.2) | 394 (7.5) | <0.0001 |
| Grade, n (%) |  |  |  |
| Well/Moderately | 236 (81.9) | 3400 (65.7) |  |
| differentiated |  |  |  |
| Poorly differentiated | 52 (18.1) | 1774 (34.3) | <0.0001 |
| Tumor size, n (%) |  |  |  |
| ≤2 cm | 236 (81.9) | 3400 (65.7) |  |
| >2 cm | 52 (18.1) | 1774 (34.3) | <0.0001 |
| Axillary node involvement, n (%) | |  |  |
| Negative | 177 (58.6) | 2922 (56.1) |  |
| Positive | 125 (41.4) | 2290 (43.9) | 0.39 |
| ER status, n (%) |  |  |  |
| Negative | 52 (17.2) | 1369 (26.6) |  |
| Positive | 250 (82.8) | 3778 (73.4) | 0.0003 |
| PR status, n (%) |  |  |  |
| Negative | 87 (29.3) | 1910 (38.9) |  |
| Positive | 210 (70.7) | 3002 (61.1) | 0.001 |
| HER2 status, n (%) |  |  |  |
| Negative | 214 (89.5) | 3166 (79.1) |  |
| Positive | 25 (10.5) | 837 (20.9) | <0.0001 |
| P-values were calculated using the chi-squared test. | | |  |
| Abbreviations: E-cad, E-cadherin; ER, estrogen receptor; PR, progesterone receptor | | | |

| **Supplemental Table 6.** Case-case analyses of established breast cancer risk factors with E-cadherin tumor tissue expression (low/high) stratified by tumor histology among estrogen receptor (ER)-positive tumors, using a score of 0 to define E-cadherin loss | | | | | | | | | | | | | |
| --- | --- | --- | --- | --- | --- | --- | --- | --- | --- | --- | --- | --- | --- |
|  | **ER-positive Tumors** | | | | **Lobular Tumors** | | | |  | **Ductal/Mixed Tumors** | | | |
|  |  | **Cases, N** |  |  |  | **Cases, N** |  |  |  |  | **Cases, N** |  |  |
| **Risk Factor** | **No. Studies** | **(E-cadherin Low/High)** | **OR (95% CI)^a^** | **P-het^b^** | **No. Studies** | **(E-cadherin Low/High)** | **OR (95% CI)^a^** | **P-het^b^** |  | **No. Studies** | **(E-cadherin Low/High)** | **OR (95% CI)^a^** | **P-het^b^** |
| **Family history of breast cancer** | 10 |  |  |  | 9 |  |  |  |  | 10 |  |  |  |
| Absent |  | 159/2262 | 1.0 (Ref) |  |  | 107/275 | 1.0 (Ref) |  |  |  | 48/1681 | 1.0 (Ref) |  |
| Present |  | 63/908 | 1.00 (0.70-1.41) | 0.98 |  | 39/137 | 0.64 (0.39-1.08) | 0.09 |  |  | 21/689 | 1.20 (0.66-2.20) | 0.56 |
| **Age at menarche** | 10 |  |  |  | 7 |  |  |  |  | 10 |  |  |  |
| ≤12 years |  | 48/826 | 0.92 (0.63-1.33) |  |  | 32/114 | 0.98 (0.58-1.65) |  |  |  | 15/636 | 0.68 (0.35-1.32) |  |
| 13 years |  | 53/599 | 1.27 (0.89-1.82) |  |  | 40/80 | 1.76 (1.05-2.96) |  |  |  | 12/460 | 0.77 (0.38-1.55) |  |
| ≥14 years |  | 89/1110 | 1.0 (Ref) | 0.25 |  | 56/157 | 1.0 (Ref) | 0.06 |  |  | 29/834 | 1.0 (Ref) | 0.49 |
| **Parity** | 9 |  |  |  | 8 |  |  |  |  | 9 |  |  |  |
| Nulliparous |  | 34/453 | 0.99 (0.66-1.48) |  |  | 24/55 | 1.37 (0.77-2.42) |  |  |  | 10/329 | 1.05 (0.51-2.19) |  |
| 1 |  | 39/579 | 0.84 (0.57-1.23) |  |  | 30/79 | 1.23 (0.73-2.08) |  |  |  | 8/425 | 0.60 (0.27-1.34) |  |
| ≥2 |  | 120/1656 | 1.0 (Ref) | 0.65 |  | 78/246 | 1.0 (Ref) | 0.49 |  |  | 38/1239 | 1.0 (Ref) | 0.42 |
| **Age at first birth (among parous women)** | 7 |  |  |  | 6 |  |  |  |  | 7 |  |  |  |
| <20 years |  | 11/140 | 1.0 (Ref) |  |  | 6/15 | 1.0 (Ref) |  |  |  | 5/106 | 1.0 (Ref) |  |
| 20-24 years |  | 51/713 | 0.85 (0.43-1.69) |  |  | 37/110 | 1.04 (0.36-3.02) |  |  |  | 14/539 | 0.53 (0.18-1.52) |  |
| 25-29 years |  | 39/482 | 0.98 (0.48-1.99) |  |  | 20/70 | 0.81 (0.26-2.49) |  |  |  | 17/367 | 0.93 (0.33-2.63) |  |
| ≥30 years |  | 12/282 | 0.54 (0.23-1.27) | 0.57 |  | 8/36 | 0.77 (0.21-2.76) | 0.84 |  |  | 4/222 | 0.36 (0.09-1.42) | 0.17 |
| **Oral contraceptive use (among women at age ≤50 years^c^)** | 5 |  |  |  | 4 |  |  |  |  | 5 |  |  |  |
| Never |  | 18/288 | 1.0 (Ref) |  |  | 9/39 | 1.0 (Ref) |  |  |  | 8/221 | 1.0 (Ref) |  |
| Ever |  | 24/384 | 1.77 (0.82-3.81) | 0.14 |  | 14/41 | 2.77 (0.77-10.0) | 0.12 |  |  | 9/310 | 1.18 (0.35-4.01) | 0.79 |
| **Any menopausal hormone (MH) use (among women at age >50 years^c^)** | 7 |  |  |  | 5 |  |  |  |  | 7 |  |  |  |
| Never |  | 61/852 | 1.0 (Ref) |  |  | 46/125 | 1.0 (Ref) |  |  |  | 14/632 | 1.0 (Ref) |  |
| Ever |  | 63/687 | 1.57 (1.06-2.33) | 0.02 |  | 47/113 | 1.30 (0.77-2.20) | 0.33 |  |  | 13/482 | 1.38 (0.61-3.11) | 0.44 |
| **Type of ever MH use (among women at age >50 years^c^)** | 7 |  |  |  | 5 |  |  |  |  | 7 |  |  |  |
| Never |  | 61/852 | 1.0 (Ref) |  |  | 46/125 | 1.0 (Ref) |  |  |  | 14/632 | 1.0 (Ref) |  |
| Estrogen only |  | 8/81 | 1.79 (0.77-4.17) |  |  | 5/16 | 0.96 (0.29-3.13) |  |  |  | 2/51 | 1.97 (0.38-10.1) |  |
| Estrogen + Progestin |  | 10/158 | 1.24 (0.60-2.58) |  |  | 7/22 | 1.25 (0.47-3.31) |  |  |  | 3/113 | 1.29 (0.33-4.97) |  |
| Unknown |  | 45/448 | 1.66 (1.06-2.61) | 0.12 |  | 35/75 | 1.40 (0.76-2.56) | 0.72 |  |  | 8/318 | 1.32 (0.50-3.49) | 0.84 |

| ^a^Logistic regression analyses were used to estimate the associations between E-cadherin tumor tissue expression and established breast cancer risk factors using E-cadherin expression levels (low vs. high) as the outcome variable and the risk factors as the independent variable, adjusted for age (10-year categories) and study site. |  |  |  |  |  |  |  |
| --- | --- | --- | --- | --- | --- | --- | --- |
| ^b^P-values for heterogeneity by E-cadherin subtype were estimated using global F test, adjusted for age and study site.  ^c^Age ≤50 years was used as a proxy for premenopausal status and age >50 years was used as a proxy for postmenopausal status. |  |  |  |  |  |  |  |
| Abbreviations: OR=odds ratio, CI=confidence interval, ER=estrogen receptor | |  |  |  |  |  |  |
| **Note:** For each variable, the category that has been shown to be associated with the lowest overall breast cancer risk in the literature was selected as the reference category. The case-case OR may be interpreted as the ratio of case-control ORs for E-cadherin low tumors (vs. controls) and E-cadherin high tumors (vs. controls). The case-case OR >1 may suggest that the risk factor association is more strongly associated with E-cadherin low tumors than with E-cadherin high tumors (OR_E-cadherin low vs. control_ > OR_E-cadherin high vs. control_). |  |  |  |  |  |  |  |
